# Dr. William H. Stokes, of the Mount Hope Institution, near Baltimore, Md., and the American Journal of Insanity

**Published:** 1849-02

**Authors:** 


*Dr. William H. Stokes, of the Mount Hope Institution, near Baltimore, Md., and the American Journal of Insanity.*

We cheerfully give insertion to the following article contained in the last number of the American Journal of Insanity, occasioned by a letter from Dr. Stokes, which was transmitted to us as well as to the Editor of that Journal, and which appeared in the number of this Journal for December, 1848. ED. BUFFALO MED. JOURNAL.

In the April number of the Journal of Insanity, 1848, the Editor gave a brief account of several Institutions for the insane that he visited during an excursion to the South. He thus mentions his visit to the Mount Hope Institution.

"The Mount Hope Hospital is but a short distance from Baltimore; it belongs to the "Sisters of Charity," and is wholly under their management. Dr. Stokes, of Baltimore, visits it daily. He was at the hospital when we called, and with one of the Sisters accompanied us through the entire establishment, which we found very neat and in good order. The number of insane was about sixty, three-fourths of whom were women, This hospital also receives cases of *mania* a potu. The institution is defective in many respects, especially as to proper means of heating and ventilation; in facilities for affording labor to patients, and we should also say in *Medical Supervision*. Dr. Stokes is well qualified, we believe, for the care of the insane, but he is, we understand, only hired to visit the institution daily and prescribe for such patients as the Sisters request. In his late Report he dwells at considerable length on the great importance of medical treatment in insanity, and apprehends Medical Officers of insane institutions have neglected too much the resources of medicine. This may be correct, but if so, how important it is that a household of insane persons should be under the supervision of a Resident Physician, who can vary the treatment according to circumstances. Every one experienced in the care of the insane well knows, that at no other time of the twenty-four hours, are the services of a physician more essential in a Lunatic Asylum than in the evening. Besides it must be difficult to establish a uniform and good *system* of moral treatment, unless the selection, instruction and discharge of the attendants on patients, are entirely in the hands of the Medical Officer.

As we have said, the Hospital as to neatness, was in excellent order, and we could not but admire that self-sacrificing spirit of the pious and benevolent females who have charge of it. They are we believe most faithful and excellent nurses."

It must be evident to every unprejudiced mind, that the foregoing remarks were dictated by kind and respectful feelings towards Dr. Stokes, and the proprietors of the Institution. But to our surprise Dr. Stokes thinks differently; and in October last sent us the following letter, which we cheerfully publish, and as early as possible.

[The letter follows which is the same communicated for this Journal, Dec., 1848, and is accordingly omitted.] EDITOR BUFF. JOUR.

STATE LUNATIC ASYLUM, UTICA, Dec. 4, 1848.

To Dr. Stokes:—

DEAR SIR:—Your letter was not received until the last number of the *Journal* was published, but I hasten to give it as early an insertion as is practicable.

I am much surprised that you complain of what I said respecting the Mount Hope Institution. The defects to which I but briefly alluded, respecting the warming and ventilation, were so obvious, and are so strongly condemned by conductors of similar establishments, and by all good authority on such subjects, that I did not suppose that I was saying anything but what would be in accordance with your own views, and serve to strengthen your exertions for improvement. Part of an Institution in New England, of which I had charge for several years, and a small portion of this Asylum were formerly warmed, as your old wing and Lodge now are, by *close stoves*; and I know by experience, that where they are used, the air cannot be kept pure. I always considered them objectionable, and felt obliged to any one for condemning them, and thus aiding me in effecting their removal. The close stoves in your old wing and in your Lodge, where your most filthy and most deranged class of patients are kept, serve but to heat the impure and fetid air of their apartments, instead of furnishing to them, as they should have, a regular supply of fresh air, moderately heated. Even your hot-air furnaces in your new wing and centre, heated by coal, though far better than your stoves, are objectionable in such establishments, and are so considered by all good authority, and have been abandoned in England and are disappearing in this country. Of this you do not seem to be aware. Thus you say our "arrangements for ventilation and warmth are unsurpassed by any similar establishment in this country or in Europe." Now Dr. Bell of the McLean Asylum, who recently visited England for the express purpose of ascertaining the modern improvements in institutions for the insane, says in his published report:—"The hot-air furnace *is universally abandoned in Great Britain, in this class of Institutions.* Under none of its thousand modifications did it meet *certain great and obvious objections* which rendered its employment inexpedient, where an atmosphere of a high hygienic quality is as essential as it is in an Insane Asylum."

We could have truly stated what we did of places of ventilation and warming which are far less objectionable than yours, and have no hesitation in saying, that *any Institution for the Insane, that has not some means for effecting a forced ventilation, and arrangements calculated to insure an abundant and regular supply of fresh air, heated by a method that will certainly prevent its ever being more than moderately warmed, is seriously defective.*

As regards *labor*, I also but expressed the opinion of all good authority. But you give us to understand, that your 'patients have at no period of their lives been accustomed to labor.' I judged differently from their appearance, but may have been mistaken; yet your own report informs us that you have a large number of of 'laborers,' 'farmers,' 'brick-layers,' 'servant-maids,' &c.

As respects *medical supervision*, I was also in accordance with all good authority, and with the practice of every other Institution for the insane, in this country. And do you intend to have the public understand that an Institution filled with a large number of insane persons, and patients suffering from other diseases, such as Mania a potu, Consumption, Typhus and Bilious Fevers, &c., all of which are received at the Mount Hope Institution, are just as well provided for, by having a physician who resides several miles distant and engaged in other business, hired to visit it twice a day; as it would be to have him reside in the Institution, and devote to it all his time? Surely, you will not so insult the common sense of mankind, and condemn the general practice of this and other countries. The necessity for a Resident Medical Officer for such Institutions, is everywhere acknowledged. The distinguished 'Commissioners of Lunacy' in England, in remarking upon the Norfolk Asylum, say;—'The most serious defect in this Institution, *and one which may be attended with the most mischievous, if not fatal consequences,* is the want of a Resident Medical Officer.'

On this subject of medical supervision, you accuse me with 'asserting in regard to yourself what is perfectly destitute of foundation;' but I do not find that you adduce the least evidence of so rude and serious a charge. I said, 'We understand Dr. Stokes is only hired to visit the Institution daily, and prescribe for such patients as the Sisters request.' We certainly did so understand at the time of our visit; and is it not strictly true? Mount Hope Institution is a private establishment,—you are not its owner or manager, and have no authority there but what you derive from the Sisters who hire you, consequently, what I said must be correct.

You admit that you have not the selection and control of the attendants on patients, but say, 'the sisters constitute the corps of attendants.' Are they the attendants upon the male patients?

You seem to rely much upon the circumstance that you report but few deaths. You must certainly know there can be no just comparison of a private Institution that receives only such patients as its proprietors choose, and can discharge them when they please—with those that are obliged to receive all sent to them, and even to give the preference to the worst cases, and obliged to keep such until they die.

An Institution may be very badly managed, and yet report but few deaths; as those not likely to live are removed before death. To what extent this is the practice at Mount Hope Institution we know not; but receiving as it does, most of its patients from the immediate vicinity, and those belonging to the wealthy or non-laboring class as you intimate, it would seem very probable, and *we think very proper*, that previous to death, they are, as your report phrases it, 'removed by friends.' That something of this is done, is evident from your Reports. Thus, certain cases of consumption, organic diseases of the heart,—diseases sure to prove fatal, we see were removed 'unimproved,' and in a few days after admission. Thus, a case of consumption received the third of June was discharged on the tenth, 'in the last stage of phthisis,'—a case of organic disease of the heart, in twelve days after admission 'unimproved,'—and a case of typhus fever discharged the next day 'unimproved,' though a case received the same day was retained and recovered. Besides, I notice some mistakes. Thus, in your table of deaths, only five are given, but in your Register seven may be found. I also see a considerable number of cases denominated insanity, that we believe would not be so called elsewhere. Thus, what you call Hysterical Mania, and Mania of only one or two weeks continuance, are not, correctly speaking, we apprehend, cases of insanity.

I do not see that there is anything else in your letter requiring notice from me. It is evident that we do not agree as to what constitutes a good system of warming and ventilation: but I am confident that we shall not disagree if you will read Bell, Wyman, Reid, and other late authorities on these subjects; or if you will visit some of the new Institutions for the insane at the North, where improved methods to a greater or less extent have been adopted. But if you still think all these authorities are wrong, and that the universal practice of having a resident physician in such Institutions is unnecessary, I will most cheerfully insert your facts and reasonings on these subjects in this Journal if you will send them to me:—but I beg you to recollect, that something besides *assertions* are wanted,—that your *ipse dixit*, or mine, unsupported by facts, or in contradiction to the scientific and well established improvements of the age, are worth but little.

Having thus, I am confident, most clearly shown that you have not the least ground for complaining of what I said, I might very properly close;—but your letter asserting that "Mount Hope Institution is wanting in nothing that can contribute to the recovery or promote the comfort of its patients," an assertion, that, in our opinion, cannot be truly said of any other Institution in this or any other country; has induced me to look rather carefully into your published Reports, and I am so convinced, that the welfare of that Institution and those who patronise it, and the usefulness of other Institutions that are now or may be hereafter established in this country for the insane, may be promoted by a few additional suggestions, that I feel it my duty to make them. And I do so, I beg you to believe, with none other than the most friendly feelings towards yourself and the Institution with which you are connected, and solely from a desire of promoting the welfare of that unfortunate class of our fellow beings to whom much of my life has been devoted.

For the remarks which follow, the reader is referred to the Am. Journal of Insanity. ED. BUFF. JOUR.

